# A comparison of the efficacy and safety of traditional Chinese medicine external treatment for the hyperemesis gravidarum: A protocol for systematic review and network meta-analysis

**DOI:** 10.1097/MD.0000000000023019

**Published:** 2020-11-06

**Authors:** Rugen Yan, Junyi Zhan, Gongxue Liu, Changzhong Li, Pingping Cai, Yin Chen, Huanze Cao

**Affiliations:** a The First Clinical College, Shandong University of Traditional Chinese Medicine; b Shandong Provincial Hospital Affiliated to Shandong First Medical University, Jinan, Shandong Province; c Xuyi County Hospital of Chinese Medicine, Xuyi, Jiangsu Province, China.

**Keywords:** hyperemesis gravidarum, network meta-analysis, protocol, systematic review, traditional Chinese medicine external treatment

## Abstract

**Background:**

The symptoms of frequent nausea and vomiting, inability to eat, and fluid imbalance caused by hyperemesis gravidarum (HG) seriously impact the quality of life of pregnant women. In some serious cases, it is even necessary to terminate the pregnancy, and threatens the life of the pregnant woman. A great many of studies have proved that HG can be effectively treated by traditional Chinese medicine (TCM) external treatment. Nevertheless, its relative effectiveness and safety haven’t yet been confirmed because of the variety of TCM external treatment. Therefore, we will use the method of network meta-analysis to verify the effectiveness and safety of different types of TCM external treatment for the HG.

**Methods:**

In this study, English and Chinese literatures that meet the requirements will be searched in EMBASE, PubMed, Cochrane Library, Web of Science, CnKI, VIP, Wanfang, and CBM. Regardless of whether blinding is used or not, all randomized controlled trials (RCTs) using TCM external treatment for the HG will be included. Then, Stata 16.0 will be used to conduct a series of pairwise meta-analysis. WinBUGS 1.4.3 and Stata 16.0 will be used to conduct Bayesian network meta-analysis to evaluate the relative results of different TCM external treatments for the HG. The quality included in the study will be evaluated through the classification of Grading of Recommendations Assessment, Development and Evaluation (GRADE).

**Results:**

This study will evaluate the effectiveness and safety of TCM external treatment for the HG according to the primary and secondary outcomes, and we rank different kinds of TCM external treatments in accordance with effectiveness. The primary outcomes are the intensity of nausea and vomiting. Secondary outcomes include quality of life, adverse outcome of pregnant women, adverse outcome of fetal, duration of hospitalization and so on.

**Conclusion:**

This study will provide more convincing and detailed information of TCM external treatment for the HG, and the reference value for clinical treatment.

**INPLASY registration number:**

INPLASY 202090089.

## 1 Introduction

Nausea and vomiting are the most common symptoms in the first trimester of pregnancy, affecting approximately 90% of pregnant women worldwide.^[1]^ Inside, nausea can last about 20 weeks, and even 20% of women will last longer.^[2]^ 0.3% to 3.6% of pregnant women will have more severe symptoms and usually require hospitalization, although nausea and vomiting of pregnancy are common and not serious usually.^[3]^ This is called hyperemesis gravidarum (HG) characterized by persistent, severe nausea, and vomiting during pregnancy, which can lead to dehydration, electrolyte disorders, weight loss, and ketonuria, and affect the quality of life of pregnant women seriously.^[4,5]^ The morbidity of HG varies worldwide because of diagnostic criteria and ethnic differences. However, most of studies believe that HG is more common in non-Caucasian race and non-smoking young primiparas. Worldwide, the morbidity of ethnic groups in Asia and the Middle East is relatively high.^[6]^ HG can cause an enormous financial burden. As is reported, it costed 1827 dollars to treat HG for per patient.^[7]^ As well as, many serious complications can be led by HG. An increasing number of studies^[8,9]^ have shown that HG is associated with prematurity, small for gestational age infant, nervous system retardation, and autism spectrum disorder in offspring. Besides, ACGO guidelines pointed out that Vernicks encephalopathy caused by HG is closely related to maternal death or permanent neurological dysfunction in 2018. In addition to increasing the hospitalization rate, some pregnant women decide to terminate their pregnancy because of severe psychosocial illness due to HG. Therefore, it is particularly important for the active treatment of HG to maintain maternal and infantile health.

According to the current research, the study on the pathogenesis of HG is not clear, and the treatment schemes are different in different countries. Although there are many common drugs to treat HG (such as vitamin B6, antihistamines, dopamine antagonists, benzodiazepines, serotonin, or antagonists, etc),^[10]^ many credible research results show that HG can hardly be cured by these drugs completely.^[11]^ Moreover, when Cochrane reviewed the above treatment methods in 2015, found that there was almost no evidence in the treatment of HG to support that one intervention method is better than the other.^[12]^ As a result of the concerning from pregnant women about the side effects of the drug and its teratogenic effect on the fetus, psychological resistance of them to drugs is very critical. Consequently, more and more patients begin to pay attention to the safe, effective, and non-toxic side effects TCM external treatment (such as: acupuncture, acupressure, acupoint sticking, etc). Tara et al^[13]^ compared the therapeutic effects of pressing on Neiguan (PC6) with that of vitamin B6 combined with metoclopramide through a randomized controlled trial (RCT). The results showed that pressing on Neiguan (PC6) could significantly reduce the degree and frequency of nausea and vomiting in pregnant women. In the network meta-analysis of RCT of HG, Sridharan et al^[14]^ found that the curative effect of acupuncture and acupoint pressing was better than that of other therapies, which once again proved the efficacy of TCM external treatment. In addition, the safety of TCM external treatment such as acupuncture and acupoint pressing in the treatment of HG has been confirmed by the recently published guidelines of evidence-based medicine in many countries.^[15–17]^

There have already been multiple systematic reviews to evaluate the efficacy of various TCM external treatments for HG, but the traditional meta-analysis can only evaluate 2 kinds of intervention measures and cannot directly compare >2 kinds of intervention measures. Therefore, there is an urgent need for network meta-analysis to systematically evaluate the efficacy and safety of different kinds of TCM external treatments for HG, so as to provide a better guidance for clinical practice, as well as a better protection for maternal and infant health.

## 2 Objective

The purpose of this network meta-analysis is to evaluate the effectiveness and safety of TCM external treatment for the HG.

## 3 Method

The agreement was designed in accordance with the “the Preferred Reporting Items for Systematic Review and Meta-analysis Protocols”^[18]^ and registered with INPLASY (ID=INPLASY202090089, URL https://inplasy.com/inplasy-2020-9-0089/). The results of the systematic review and network meta-analysis are expected to be published in recognized journals. Since this is a systematic literature study, there is no need to apply for ethical review.

### 3.1 Eligibility criteria

#### 3.1.1 Type of studies

Regardless of whether blinding is used or not, all RCTs of TCM external treatment for the HG will be included. Clinical trials without a control group will be excluded. The language is restricted to English and Chinese.^[19]^

#### 3.1.2 Type of participants

Pregnant women who were clinically diagnosed with HG will be included, regardless of age or race. The diagnosis of HG should meet the diagnostic criteria of the guideline for the HG established by the ACOG. Patients are not invited to participate in the writing or editing of this article for readability or accuracy.

#### 3.1.3 Interventions and comparators

The experimental group will include all type of TCM external treatments for the HG such as acupuncture, acupressure, acupoint sticking, acupoint injection, and so on. The control group was treated with antiemetic drugs or different types of TCM external treatments. Patients in both groups were treated with intervention based on routine rehydration therapy, and duration of treatment unlimited.

#### 3.1.4 Outcomes indicators

##### 3.1.4.1 Primary outcomes

The primary outcomes of what we desperately want are the intensity and severity of nausea and vomiting (frequency and duration of nausea and vomiting). The severity of nausea and vomiting was evaluated by the Modified Pregnancy-Unique Quantification of Emesis and Nausea (PUQE) which is a reliable and effective method.^[20]^ The score of the PUQE index is closely related to the quality of life.

##### 3.1.4.2 Secondary outcomes

(1) Quality of life: The physical symptoms, aggravating factors, fatigue, emotion, and limitation were scored by the pregnancy Nausea and Vomiting of Pregnancy Quality of Life questionnaire (NVPQOL).^[21]^ The lower the score, the higher the quality of life.
(2) Adverse outcomes of pregnant women: weight loss, gestational hypertension, and preeclampsia, etc.
(3) Adverse outcomes of fetal: spontaneous abortion, stillbirth, premature infants, low birth weight infants, etc.
(4) Duration of hospitalization.

### 3.2 Search strategy

We will conduct a comprehensive search in the PubMed (A search strategy is as shown in Table 1), EMBASE, Web of Science and Cochrane libraries to determine the available English data. In addition, we will also search for Chinese e-bibliographic database resources, mainly including China national knowledge infrastructure (CNKI), Weipu database (VIP), Wanfang database, and China Biology Medicine (CBM). Furthermore, we will also search for ongoing or unpublished trials such as the International Clinical Trials Registry Platform, the NIH Clinical Trails, and the Chinese Clinical Register.

**Table 1:** Search strategy for the PubMed.

NO.	Search item
#1	Hyperemesis Gravidarum[MeSH Terms]
#2	Hyperemesis gravidarum[Title/Abstract] OR Pernicious Vomiting of Pregnancy [Title/Abstract] OR Pregnancy Pernicious Vomiting[Title/Abstract]
#3	#1 OR #2
#4	Traditional Chinese Medicine External Treatment[MeSH Terms]
#5	Acupuncture[Title/Abstract] OR Acupoint Pressing[Title/Abstract] OR Acupoint Sticking[Title/Abstract] OR Acupoint Injection[Title/Abstract]
#6	#4 OR #5
#7	Randomized Controlled Trial[Publication Type]
#8	Randomized Controlled Trial[Title/Abstract] OR Randomized[Title/Abstract] OR Double-blind method [Title/Abstract] OR Randomly[Title/Abstract]
#9	#7 OR #8
#10	#3 AND #6 AND #9

### 3.3 Literature screening and data extraction

#### 3.3.1 Literature screening

First of all, we will search each database according to the established retrieval strategy, and all the literature titles will be imported into the EndNote document management software, which will automatically duplicate checking and rule out duplicate documents. Then, the checked literature will be preliminarily screened by the title and abstract. The full text of the rest are downloaded, of which they do not meet the inclusion criteria are further excluded. Finally, the selected literature was extracted back-to-back by 2 researchers. If there are inconsistencies in the information extracted by the 2 researchers, we will correct them by reading the original text again, or consult the third researcher.

#### 3.3.2 Data extraction

There will be 2 researchers extract data independently using Excel 2019. Then, these extracted information will be roughly divided into 4 types: research information (such as the first author, title, year of publication, random or blinding, etc), participant information (such as age, race, etc), intervention information (such as treatment method, course of treatment, Comparison group, etc), and outcomes (including primary and secondary outcomes). If there are disagreements in the process of information extraction, we will consult third-party researchers.

### 3.4 Assessment of risk of bias

There will be 2 reviewers to evaluate the quality of the included studies by Cochrane Handbook (version 5.2.0) independently. The evaluation scope included: random sequence generation, allocation concealment, blinding of patients and researchers, blinding of outcome evaluators, data integrity of results, selective reporting, and other biases. According to the results of each study, the included studies will be judged as “low risk,” “high risk,” and “unclear.” If there are different opinions on the evaluation results, the third researcher will participate in the discussion and make a final decision.

### 3.5 Data analysis

#### 3.5.1 Characteristics of the eligible studies

We will conduct descriptive statistics on the population characteristics of the eligible studies that including age, type of comparison, duration of illness, race, etc.

#### 3.5.2 Pairwise meta-analyses

The chi-squared test in Stata 16.0 (Stata Corporation, College Station, TX) will be used to evaluate the *P*-value between the results of each study, and *I*^2^ will be used to quantitatively determine the size of heterogeneity. If *I*^2^ < 50%, *P* > .05, it means that there is no heterogeneity between the studies, and the fixed effects model can be used; if *I*^2^ > 50%, *P* < .05, it means that there is heterogeneity between the studies, then the source of heterogeneity should be analyzed, such as age, disease course, etc. After excluding obvious heterogeneous effects, a random effects model will be adopted.

#### 3.5.3 Network meta-analyses

The mean difference (MD) will be used for measurement data and the odds ratio (OR) for enumeration data, by Stata 16.0 software. Each effect size will be given its estimated value and 95% confidence interval. We will conduct Bayesian network meta-analysis by the Markov chain Monte Carlo method in WinBUGS1.4.3, that is simulated by 4 chains.^[22]^ The original number of iterations is set to 50,000, and the effect of the initial value is eliminated by the first 20,000 times of annealing, meanwhile the last 30,000 times are used for sampling. In the iterative process, the trajectory of the fluctuation of the Markov chain Monte Carlo method is reflected and the potential scale reduced factor (PSRF) quantitative analysis method is used to diagnose the convergence of the model. The number of iterations and annealing times were adjusted according to the data characteristics of each outcome index and the PSRF value, and the area under the cumulative ranking probability (SUCRA) was used for ranking. The larger the value of SUCAR, the better the effect of the intervention.^[23]^

#### 3.5.4 Heterogeneity analyses

##### 3.5.4.1 Subgroup analyses

If the studies included are highly heterogeneous, we will conduct a subgroup analysis to explore age, race, different types of TCM external treatments, treatment time, methodological quality, etc.

##### 3.5.4.2 Sensitivity analyses

For the purpose of ensuring the credibility of the research results, we will analyze their sensitivity by the following methods:

(1) Changing of the inclusion criteria.
(2) Exclusion of low-quality or open studies.
(3) Analysis of the same data by different statistical methods/models.

#### 3.5.5 Measures for inconsistency

The node-splitting method will be used for consistency test. If there is no statistic difference, it indicates that the results of direct comparison and indirect comparison are consistent.

#### 3.5.6 Assessment of reporting bias

We will use the funnel chart as well as the Egger test and Begg test to assess reporting bias. If deviations are found, it may be related to the negative results of the literature and the low quality of the included methods.

#### 3.5.7 Evidence quality evaluation

In order to better use the results of the study to formulate guidelines, we will use the GRADE to evaluate the quality of evidence in this study.^[24]^ Although the quality of evidence is continuous, the quality of the evidence group is ultimately divided into 4 grades by GRADE: high quality, moderate quality, low quality, and very low quality. Although the literature included are all RCTs in this study, and the evidences are initially rated as high quality, the quality of such evidence may be reduced due to the following 5 factors: the risk of research bias, inconsistent research results, and indirect evidence, inaccurate results, and publication bias. In addition, there are 3 other factors that may contribute to the improvement of the quality of evidence: large effect size, dose–effect relationship, and negative bias.

## 4 Discussion

HG is a serious early pregnancy reaction, which is mainly manifested as frequent nausea and vomiting, inability to eat, fluid imbalance. It is even necessary to terminate the pregnancy and threaten the lives of pregnant women, especially in serious condition. As pregnant women are worried about the side effects of medication during pregnancy and fetal malformations, many people are seeking non-drug treatment, and the TCM external treatment is the most prominent representative. It not only achieves the purpose of treating diseases by stimulating on the body surface, but also skillfully avoids the liver first-pass effect of oral drugs. It has no gastrointestinal irritation symptoms, and effectively solves the worries of patients. Although there are many studies on the effectiveness of TCM external treatment in HG management, the evaluation and comparison of various TCM external treatments are not sufficient. Moreover, the efficacy of different treatment methods is still lacking in detailed comparison and ranking. The purpose of this study is to provide more convincing and detailed information for the TCM external treatment of HG, and to provide references for the clinical treatment of such diseases. The results of the study are expected to be published in related journals and may attract more people, including patients and their families with HG, obstetricians and gynecologists, practice guide makers, researchers, and policy makers. We will update the content required for this agreement in the future, and will supplement it by the revising date and changing instructions. Although the problems that will be arose in the course of the study have been carefully considered and solved by us, but the following problems will inevitably arise: the included literature only includes English and Chinese, which is relatively one-sided; the quality of the literature is not high and the sample size is small. All of these problems may lead to publication bias.

## Author contributions

**Conceptualization:** Rugen Yan, Changzhong Li, Pingping Cai.

**Data curation:** Junyi Zhan, Gongxue Liu, Yin Chen.

**Funding acquisition:** Pingping Cai.

**Investigation:** Rugen Yan, Junyi Zhan.

**Methodology:** Rugen Yan, Changzhong Li, Pingping Cai.

**Resources:** Rugen Yan, Junyi Zhan, Gongxue Liu, Yin Chen.

**Software:** Gongxue Liu, Huanze Cao.

**Supervision:** Changzhong Li, Pingping Cai.

**Writing – original draft:** Rugen Yan, Junyi Zhan.

**Writing – review & editing:** Rugen Yan.
